# Evaluating the effect of recombinant human growth hormone treatment on sleep-related breathing disorders in toddlers with Prader–Willi syndrome: a one-year retrospective cohort study

**DOI:** 10.1186/s12887-023-04513-0

**Published:** 2024-01-10

**Authors:** Haiyan Guo, Jinrong Fu, Yufeng Zhou, Feihong Luo, Ruoqian Cheng

**Affiliations:** 1 Institute of Pediatrics, Children’s Hospital of Fudan University, National Children’s Medical Center, and the Shanghai Key Laboratory of Medical Epigenetics, International Co-Laboratory of Medical Epigenetics and Metabolism, Ministry of Science and Technology, Institutes of Biomedical Sciences, Fudan University, Shanghai, 200032 China; 2 Department of Endocrinology and Inherited Metabolic Diseases, National Children’s Medical Center, Children’s Hospital of Fudan University, Shanghai, 201102 China; 3 National Health Commission (NHC) Key Laboratory of Neonatal Diseases, Fudan University, Shanghai, 201102 China; 4 Department of General Medicine, National Children’s Medical Center,Children’s Hospital of Fudan University, Shanghai, 201102 China

**Keywords:** Sleep-related breathing disorders, Recombinant human growth hormone treatment, Prader–Willi syndrome, Toddlers

## Abstract

**Background:**

Recombinant human growth hormone (rhGH) therapy is beneficial for children with Prader–Willi syndrome (PWS) in improving short stature and metabolism, but the effect of early rhGH treatment on respiratory and sleep parameters for PWS children under three years old remains elusive. Thus, this study aimed to investigate the impact of rhGH treatment on sleep-related breathing disorders (SRBDs) for toddlers with PWS.

**Methods:**

A total of 17 age-matched PWS patients receiving rhGH treatment (rhGH group) and 17 control individuals not receiving rhGH treatment (non-rhGH group) were recruited for this study between October 2018 and January 2023. Data related to polysomnography-polygraphy (PSG) and serum levels of insulin-like growth factor (IGF-1) and insulin-like growth factor binding protein 3 (IGFBP-3) were collected.

**Results:**

The mean age in the rhGH group was 20.76 ± 9.22 months, which was comparable to that of the non-rhGH group (25.23 ± 13.81 months). The demographic and anthropometric parameters were similar across the two groups after 52 weeks of treatment. Administration of rhGH to toddlers did not exert adverse effects on the obstructive apnea–hypopnea index (OAHI), central apnea index (CAI), oxygen desaturation index (ODI), mean percutaneous oxygen saturation (SpO_2_), lowest SpO_2_, duration when SpO_2_ is lower than 90%, or proportion of the patients with SpO_2_ lower than 90%. Furthermore, the increased IGF-1 z-score and IGFBP-3 level did not worsen SRBDs.

**Conclusion:**

Treatment with rhGH for 52 weeks on young toddlers with PWS showed no deleterious effects on SRBDs. This shed more light on the importance of initiating rhGH therapy early in PWS patients.

**Supplementary Information:**

The online version contains supplementary material available at 10.1186/s12887-023-04513-0.

## Introduction

Prader-Willi syndrome (PWS) is a rare genetic disease that affects approximately 1 in 15 000 children [1]. Its phenotypes are expressed as 65–75% absent paternally expressed imprinted genes at 15q11.2-q13 through paternal deletion of this region, 20–30% maternal uniparental disomy (mUPD) of chromosome 15, and 1–3% an imprinting defect [1]. Patients with PWS at the infancy stage presented with low birth weight, neonatal hypotonia, difficulty in feeding, and endocrine disorders. While early diagnosis is possible with advancements in science and technology, there is currently no established cure for the disease. However, an integrated multidisciplinary approach, including the use of the synthetic version of the recombinant human growth hormone (rhGH), development therapy, and occupational therapy, is recommended to minimize complications, improve quality of life, and increase life expectancy [2].

Most children with PWS have growth hormone (GH) deficiency, which can be identified through real-time monitoring of daily spontaneous GH release and associated stimulation tests [3]. Studies have shown that rhGH therapy can improve linear growth, body composition, physical strength, agility, and mental development in children with PWS [4, 5]. This treatment should be accompanied by dietary, environmental, and lifestyle interventions for genetically-confirmed PWS patients [3]. Consensus guidelines suggest initiating rhGH treatment before the age of two [3]. However, some endocrinologists argue for initiating treatment as soon as PWS is diagnosed, especially during infancy and toddlerhood [6, 7]. It is worth noting that the initial weeks of rhGH treatment may lead to worsened sleep-related breathing disorders (SRBDs) and adenoid hypertrophy, possibly due to high levels of insulin-like growth factor 1 (IGF-1) after starting rhGH [8]. SRBDs are common symptoms of PWS, and they can contribute to poor physical health, neurocognitive function, and prognosis due to obesity, narrowed upper airways, reduced saliva excretion, adenoid/tonsillar hypertrophy, hypotonic breathing muscles, or scoliosis [9].

A study by Pacoricona Alfaro investigated the causes of death in PWS and found that out of 104 deaths, 14 patients had previously used or were currently using rhGH at the time of their death, with respiratory infection, cardiac failure, and sudden death being the main causes [10]. Moreover, a striking 98% of the patients older than 18 years old were identified as obese. In contrast, among pediatric PWS population under the age of 18, only 25% exhibited obesity [10]. Another pharmaceutical company reported that 5 out of 675 children treated with GH died suddenly due to respiratory issues [11]. Extensive research has been conducted over the past two decades to assess the safety of rhGH in PWS, and regular PSG and adenotonsillar examinations are recommended for long-term monitoring of children on rhGH treatment [12, 13]. Although the mortality rate associated with rhGH treatment in children with PWS is extremely low, there are still concerns among clinicians regarding its safety based on previous studies reporting adverse effects. There is limited research on the safety of rhGH in infants and toddlers with PWS. Therefore, the safety of rhGH therapy in infants and toddlers with PWS requires further investigation. This retrospective cohort study aims to explore the effects of initiating rhGH on SRBDs in toddlers with PWS. The article follows Strengthening the Reporting of Observational studies in Epidemiology (STROBE) checklists [14].

## Subjects and methods

### Study design

**Fig. 1:**
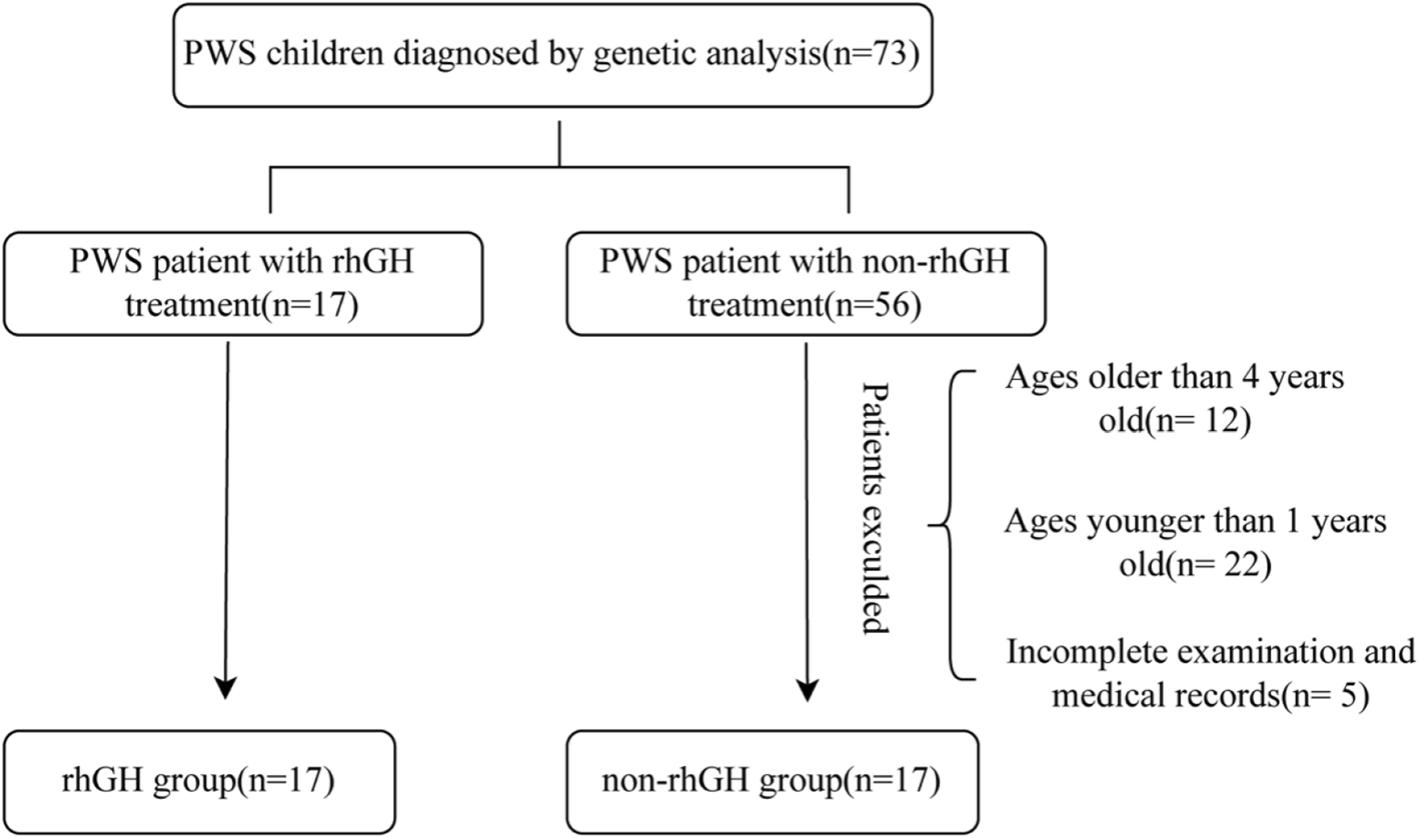
The flowchart of the enrollment process

This retrospective cohort study consisted of two groups: the rhGH treatment group and non-rhGH treatment groups, serving as controls. Participants in the rhGH treatment group were enrolled in a prospective clinical trial registered at Clinical Trials. Gov with the identifier NCT-3554031. They received rhGH treatment after being diagnosed with PWS at the Department of Endocrinology, Children’s Hospital of Fudan University. The inclusion and exclusion criteria were described in the previous paper [4]. Participants in the non-rhGH treatment group were diagnosed with PWS, who never used rhGH treatment. The inclusion criteria in the non-rhGH group were as follows: patients with genetically diagnosed PWS who had not received rhGH treatment since birth due to financial constraints, delayed diagnosis or their parents' unwillingness to use it, and whose age matched that of the rhGH group after one year of treatment. Patients with incomplete medical records were excluded from the non-rhGH group, resulting in the exclusion of 39 patients from this study. The detailed enrollment process is depicted in Fig. 1. Ultimately, a total of 34 patients were included in this study, with 17 patients in the rhGH group (Jintropin®, GeneScience Pharmaceuticals, Changchun, China) receiving rhGH treatment for 52 weeks. All subjects underwent follow-up assessments at baseline, as well as at 26 and 52 weeks after initiation of treatment. Subjects in the rhGH treatment group underwent PSG assessment thrice: before rhGH treatment, at 26 weeks, and at 52 weeks after treatment. Moreover, the patients in the rhGH group initiated rhGH treatment once they were diagnosed with PWS(median age was 4 months), and most of them were younger than 1 year when they used rhGH treatment. Participants in the non-rhGH treatment group underwent PSG assessment upon diagnosis of PWS. The demographic and anthropometric characteristics and PSG assessment results were compared between the groups. Additionally, the anthropometric characteristics and PSG assessment results of the participants in the rhGH group were compared before and after treatment.

### Demographic and anthropometric characteristics

Demographic and anthropometric characteristics, including sex, age, weight, height/length, and weight/height z-score(W/H z-score) were collected in this study. The W/H z-score was calculated using age- and sex-specific growth curves based on the World Health Organization (WHO) guidlines [15]. Overweight was defined as a W/H z-score greater than 2, while obesity was defined as a W/H z-score greater than 3 [16]. All enrolled participants underwent overnight PSG assessment. A standardized procedure was followed for clinical workups, focusing on common complications associated with Prader-Willi syndrome (PWS), such as epilepsy, scoliosis, secondary thyroid/adrenal insufficiency, as well as glucose and insulin metabolism.

### Genetic analysis

Genomic DNA was extracted from peripheral blood, and the methylation status of the patients was analyzed using methylation-specific polymerase chain reaction (MS-PCR). Patients exhibiting abnormal methylation patterns underwent further testing to explore genetic defects. Methylation-specific multiplex ligation-dependent probe amplification (MS-MLPA) was utilized to differentiate between deletion in 15q11-q13, maternal uniparental disomy (mUPD) and imprinting defect (ID) [17].

### rhGH treatment

The participants in the rhGH group were administered a dose of 0.5 mg/m^2^/day for the initial 4 weeks, which was subsequently increased to 1 mg/m^2^/day for up to 52 weeks following the initiation of treatment. All participants were diligently followed up at baseline, as well as at weeks 26 and 52 after the commencement of treatment. The final follow-up assessment was conducted on *January 18th, 2023.*

### Laboratory tests

Blood samples were obtained at 8:00 a.m. following an overnight fast. Serum levels of IGF-1 and insulin-like growth factor binding protein 3 (IGFBP-3) were measured using routine laboratory assays (IMMULITE® 2000 immunoassay system (SIEMENS, Germany)). The z-score of IGF-1 was calculated using age- and sex-specific growth curves established in a previous study conducted by Cao et al [18].

### Polysomnography-polygraphy (PSG) assessments

PSG (EMBLETTA MPR, America) assessments were performed, and various parameters were recorded, including oronasal airflow, thoracic and abdominal movements, percutaneous oxygen saturation (SpO_2_) and body positions. SRBDs especially for obstructive sleep apnea (OSA), were scored based on the recommendation from The American Academy of Sleep Medicine’s (AASM) Manual for the Scoring of Sleep and Associated Events [19]. The PSG measurements and scoring were conducted by a single experienced clinician. Several parameters were simultaneously recorded, including the obstructive apnea–hypopnea index (OAHI), central apnea index (CAI), oxygen desaturation index (ODI), and an average OAHI or CAI of more than 1 event per hour with a minimum SpO_2_ nadir of 92% during sleep was considered abnormal [20]. OSA was defined as OAHI ≥ 1.5 and was further classified into different severity levels: mild (OAHI ≥ 1.5 to < 5), moderate (OAHI ≥ 5 to < 10), and severe (OAHI ≥ 10) [21, 22].

### Statistical analysis

We performed statistical analyses using IBM SPSS (version 25.0, Chicago, IL) software and R-language with ggplot2, reshape2, ggsignif and RColorBrewer packages (version 3.6.3). Normally distributed continuous variables were presented as mean ± standard deviation, while discrete data were presented as counts and proportions. Continuous variables were assessed using a t-test, and categorical variables were compared using the χ^2^ test or Fisher’s exact test. Non-parametric tests were utilized for data with non-normal distribution. The association between parameters was performed using linear regression and multivariate logistic regressions. *P*-values < 0.05 were considered statistically significant.

## Results

### Genotype, demographic and anthropometric characteristics

A total of 34 cases of PWS patients, all under three years of age, were included in this study. Genetic results showed paternal 15q11-13 deletions in 24 (70.59%) patients and aberrant methylation (mUPD and imprinting defect) in 10 (29.41%) patients (Table 1). Amongst the 17 patients receiving rhGH treatment, 13 (76.47%) presented with paternal 15q11-13 deletions and 4 (23.53%) with aberrant methylation. Meanwhile, in the control group, genetic analysis showed that 11 cases (64.71%) were paternal deletion, and 6 were aberrant methylation. The genotypes across both groups were similar(*p* = 0.71). Moreover, for the patients with paternal deletion, there are 11 in the non-rhGH group and 13 in the rhGH group. For the patients with aberrant methylation, there are 6 in the non-rhGH group and 4 in the rhGH group. No significant differences were observed between the non-rhGH and rhGH group.

**Table 1:** Demographic and anthropometric data of enrolled participants

	non-rhGH (*n* = 17)	rhGH (*n* = 17)	*p* value
Genotype			0.71
Paternal deletion (%)	11 (64.71)	13 (76.47)	
Aberrant methylation (mUPD and imprinting defect) (%)	6 (35.29)	4 (23.53)	
Sex			> 0.05
Male (%)	7 (41.12)	8 (47.06)	
Female (%)	10 (58.82)	9 (52.94)	
Age (months)	25.23 ± 13.81	20.76 ± 9.22	0.59
Height/length (m)	0.84 ± 0.13	0.85 ± 0.08	0.81
Weight (kg)	13.65 ± 5.26	11.38 ± 3.12	0.43
W/H z-score	1.08 ± 1.97	-0.45 ± 1.27	**0.02**
Obesity and overweight (%)	6 (35.29)	1 (5.89)	**0.03**
IGF1 z-score	-0.46 ± 0.68	1.74 ± 1.75	**< 0.001**
IGFBP-3 (μg/mL)	2.17 ± 1.09	3.56 ± 1.24	**0.003**

**Fig. 2:**
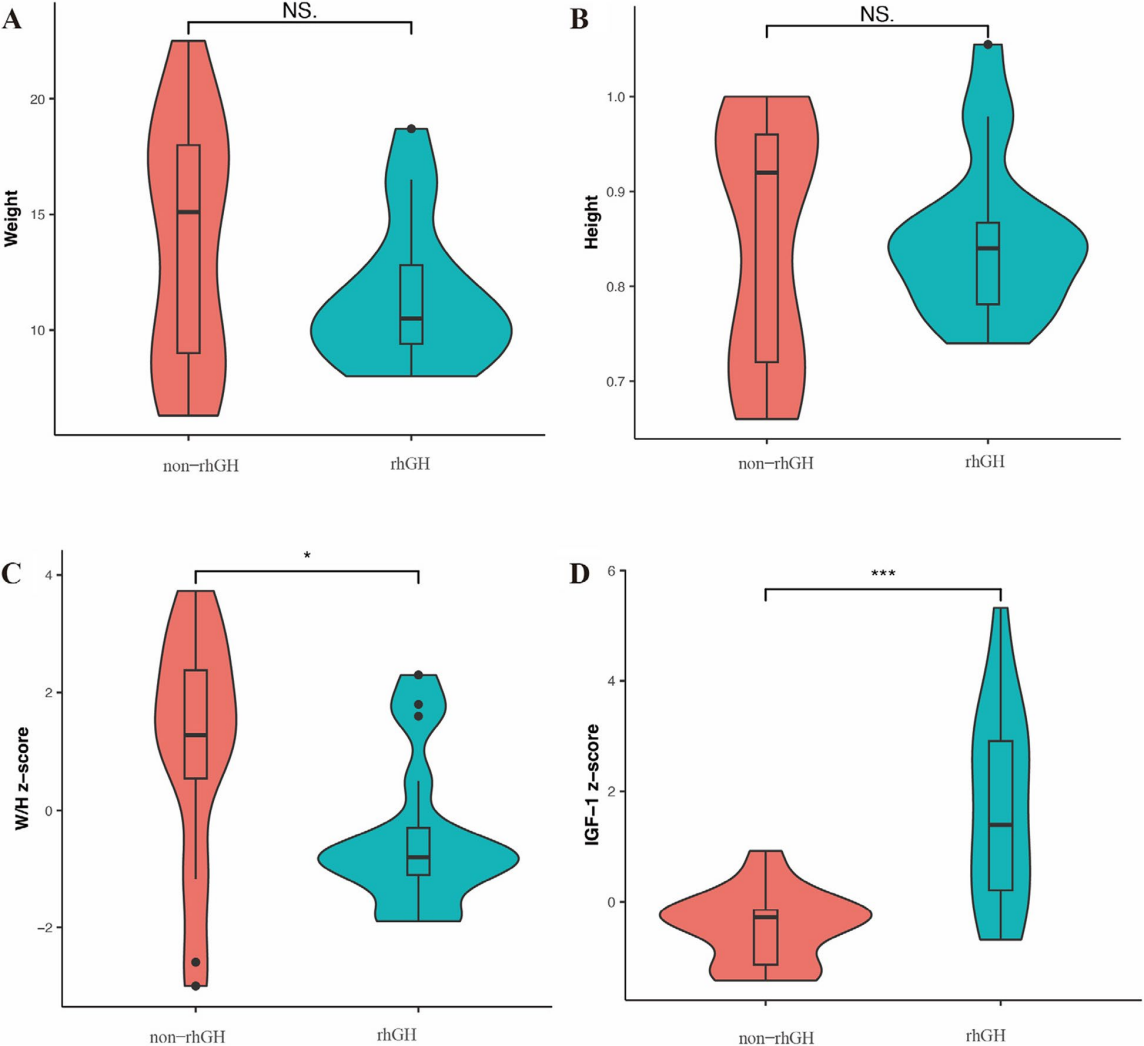
The anthropometric data and IGF-1 z-score in the non-rhGH group and rhGH group after 52 weeks treatment. No significant difference was identified for the weight (**A**) and height (**B**) between rhGH treatment and non-rhGH treatment group. The value of W/H z-score in the non-rhGH treatment group was significantly higher than the rhGH group (**C**). The level of IGF-1 z-score in the non-rhGH was significantly lower than the rhGH group (**D**). NS.: no significance, *: *p*-value < 0.05, ***: *p*-value < 0.001

We enrolled 15 males and 19 females, with 8 males and 9 females in the rhGH group, and 7 males and 10 females in the non-rhGH group. After a 52-week follow-up period for the participants in the rhGH group, we collected data related to the age, height/length, serum IGF-1 z-score and IGFBP-3 levels. The sex, age, height/length and weight did not differ across both groups. However, compared with the non-rhGH group, the distribution of height/length, height and weight/height z-score were more concentrated and the standard deviations were smaller in the rhGH group (Fig. 2*** and ***Table 1). Compared to the non-rhGH group, the value of serum IGF-1z-score and IGFBP-3 levels in the rhGH group were significantly increased (-0.46 ± 0.68 vs. 1.74 ± 1.75, *p* < 0.001; 2.17 ± 1.09 vs. 3.56 ± 1.24, *p* = 0.003). Conversely, the W/H z-score (-0.45 ± 1.27 vs 1.08 ± 1.97, *p* = 0.02) and the proportion of being obesity and overweight (1 (5.89%) vs 6 (35.29%), *p* = 0.03) significantly decreased in the rhGH group compared to the non-rhGH group (Fig. 2).

### Comparison of PSG results between non-rhGH and rhGH treatment group

**Fig. 3:**
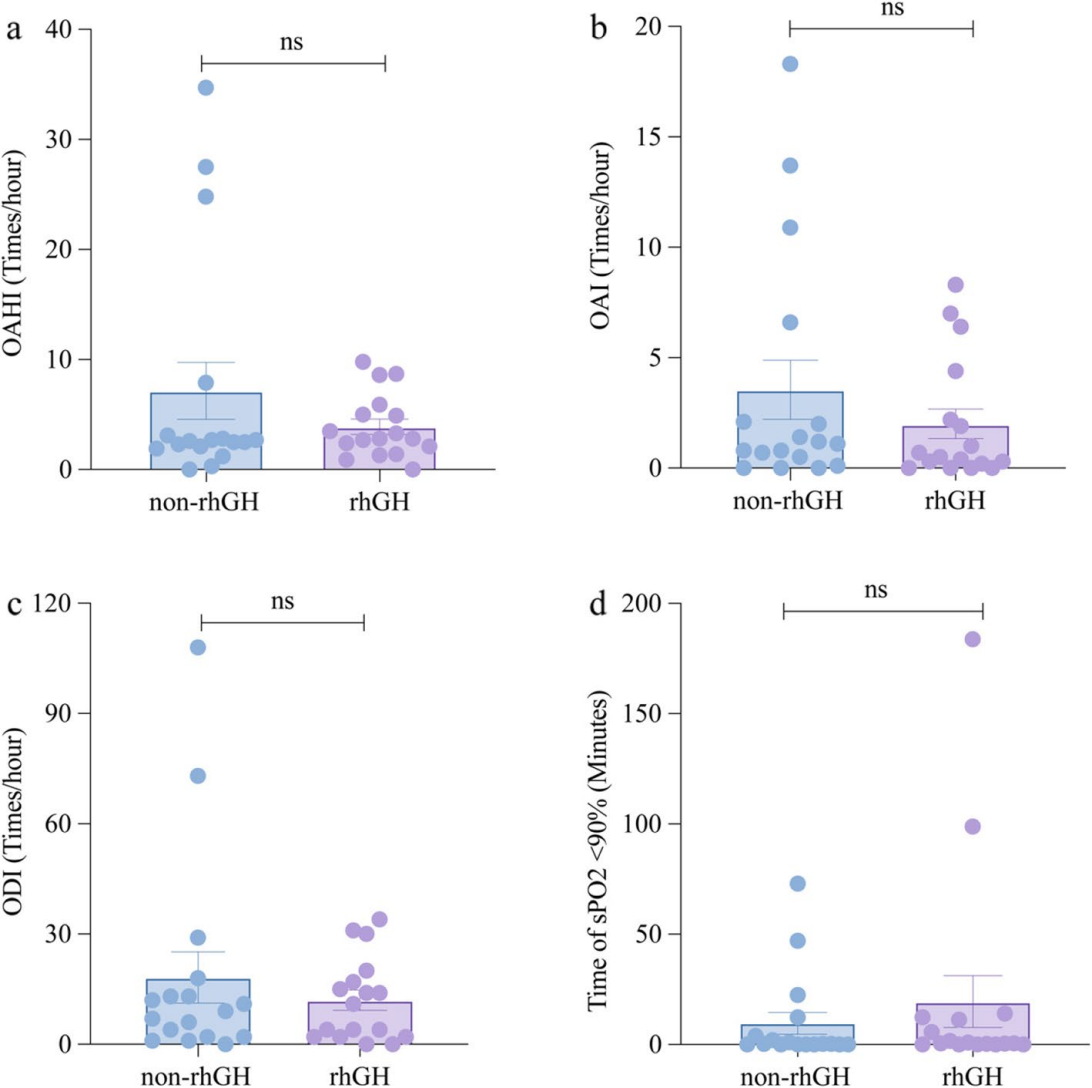
Data of the PSG assessments in the rhGH group and the non-rhGH treatment group. No significant difference was found between rhGH group and the non-rhGH group in OAHI, OAI, ODI and time of SpO2 lower than 90%. ns: no significance

PSG results are shown in Fig. 3 and Supplementary Table 1. The AHI, OAHI, CAI, ODI, mean SPO_2_, lowest SpO_2_, time of the SpO_2_ lower than 90%, and proportion of the SpO_2_ lower than 90% did not differ across both groups.

### Effects of rhGH treatment on PSG results

**Fig. 4:**
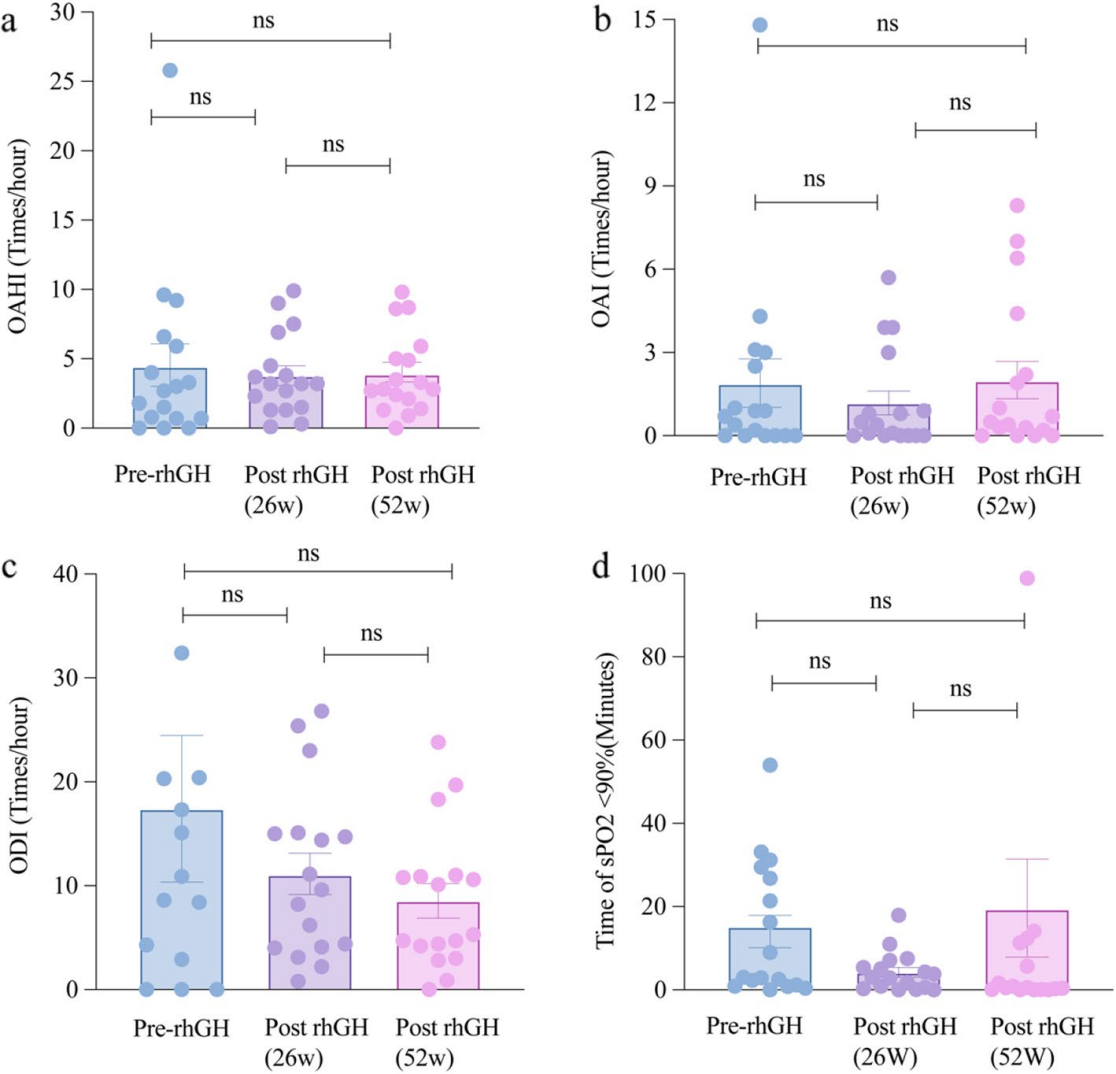
Comparsion of rhGH treatment on PSG results at the baseline, 26 weeks and 52 weeks after the treatment. No significant difference of the OAHI (**a**), OAI (**b**), ODI (**c**) and time of spO2 less than 90% (**d**) was found among the pre-treatment, 26-week and 52-week after the treatment. ns: no significance

PSG results were collected before rhGH therapy, at 26 weeks and 52 weeks post-initiation of treatment. Similarly, the PSG-associated parameters (OAHI, OAI, CAI, ODI, mean SpO_2_, lowest SpO_2_, time of SpO_2_ lower than 90%, and the proportion of SpO_2_ lower than 90%) did not differ with the treatment (Fig. 4 and Supplementary Table 2).

#### Correlation of OAHI and IGF-1 after rhGH administration

**Fig. 5:**
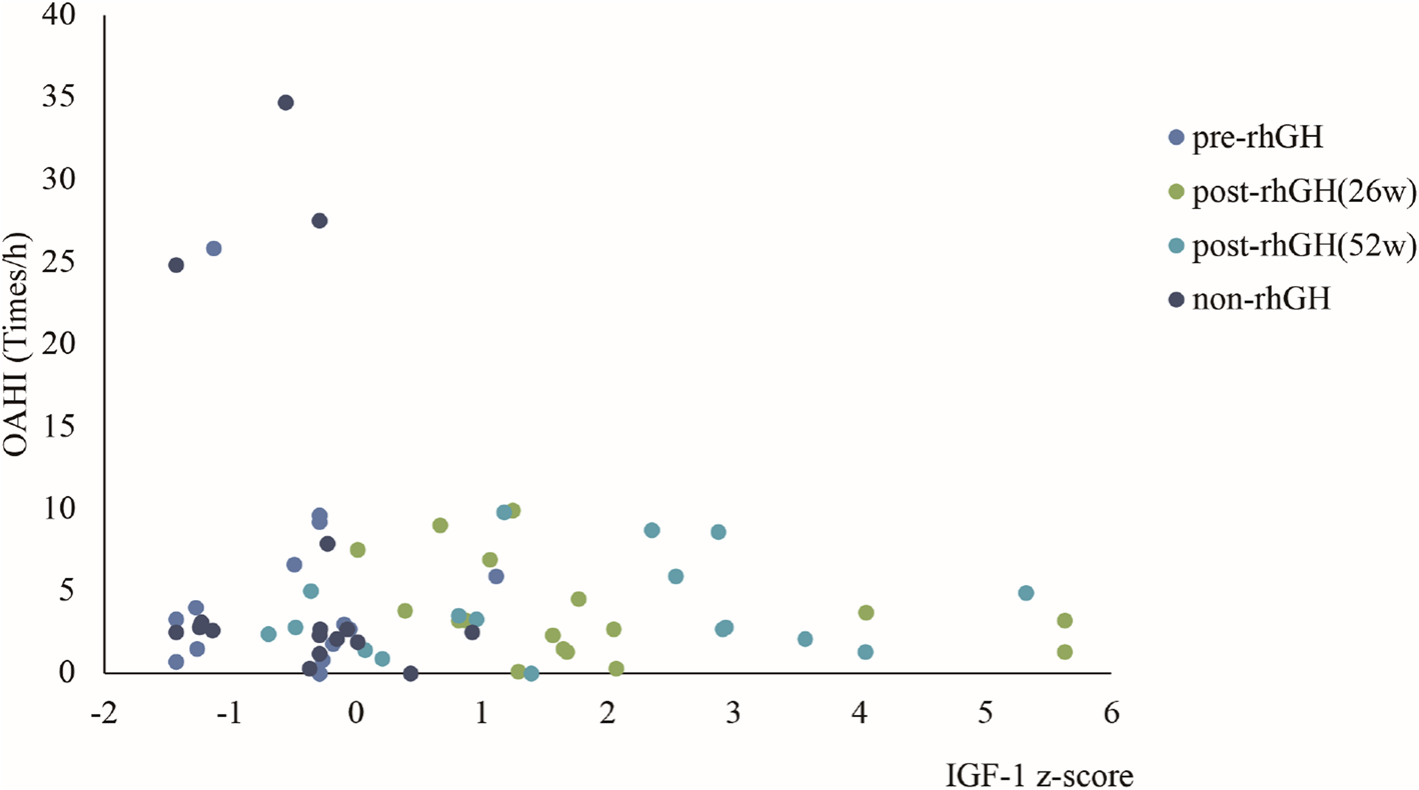
Linear regression of IGF-1 zscore and OAHI. No correlation was found between IGF-1 zscore and OAHI among the pre-rhGH, post-rhGH(26w), post-rhGH(52w) and non-rhGH groups

The serum IGF-1 level was tested among the non-rhGH treatment and rhGH treatment group before rhGH therapy, at 26 weeks and 52 weeks post-initiation of therapy. Additionally, the IGF-1 z-score was calculated by utilizing age- and sex-specific growth curves derived from Cao B’s paper [18]. Linear regression that OAHI was not correlated with IGF-1 z-score after the non-rhGH treatment and rhGH treatment (*F* = 1.65, *p* = 0.20) (Fig. 5). Similar to other research, the level of IGF-1 z-score was elevated in the rhGH group after administrating rhGH daily, but it did not exert any negative impact on OAHI.

#### Identification of non-mild OSA relevant factors

**Fig. 6:**
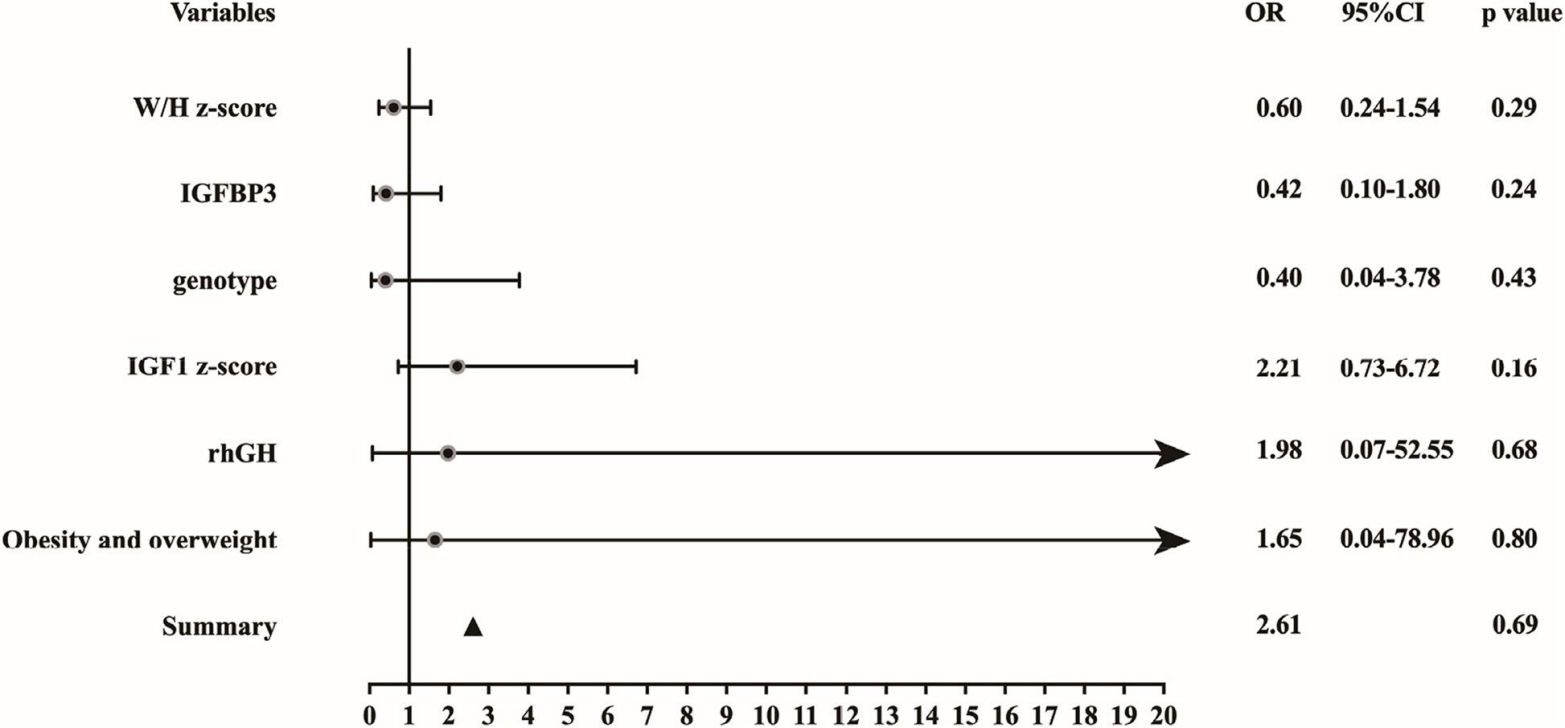
Forest plot of multi-factor logistic regression analysis of the potential factors in exacerbating OAHI in the rhGH treatment group. PWS genotype, rhGH treatment, IGF-1 z-score, IGFBP-3, obesity and overweight, and W/H z-score did not result in aggravating OAHI

Children with moderate-to-severe OAHI were excluded in this study following the inclusion criteria. However, after 1-year rhGH treatment, 4 children in the rhGH group had moderate OAHI. Figure 5 demonstrates that IGF-1 did not correlate with OAHI at the three timepoints. We performed multivariate logistic regression analysis to explore the effects of the variables on OAHI. Non-mild OSA (OAHI ≥ 5), including moderate and severe OSA, was used to identify the potentially relevant factors with rhGH treatment. We selected the parameters with p value less than 0.05 in Table 1 (W/H z-score, overweight and obesity, IGF1 z-score and IGFBP-3), as well as the genotype and rhGH treatment to perform logistic regression analysis. Multivariate logistic analysis showed these parameters were not associated with non-mild OSA. The overall *p*-value was 0.69 (Fig. 6 and Supplementary Table 3).

## Discussion

This longitudinal, retrospective cohort rhGH study analyzed a consecutive cohort of 34 children diagnosed with PWS and found that rhGH treatment exerts no negative influence on the indices of PSG evaluation. Moreover, the AHI, OAHI, CAI, ODI, mean SPO_2_, lowest SpO_2_, duration of SpO_2_ when lower than 90%, and proportion of patients with SpO_2_ lower than 90% were similar across the groups.

Lumeng et al.have shown that the prevalence of pathologically sleep-disordered breathing in patients who were not on treatment was similar to those on treatment. Moreover, the occurrence of sleep-disordered breathing was similar at six months after GH therapy onset [23, 24]. Consistent with the majority of existing literature, our findings align with these results, indicating that the occurrence of OAHI remains similar across the groups after a one-year follow-up period. These collective findings suggest that rhGH treatment is relatively safe for children under the age of three, in accordance with current research. Conversely, Zimmermann et *al*. showed that OSA increased significantly in the rhGH treatment group in the first three months [25]. Berini et al*.* showed a significant increase in the occurrence of OAHI for up to 4 years after rhGH onset, and the proportion of patients with an OAHI > 1 increased from 3 to 22, 36 and 38 at the 6 weeks, 2 years, and 4 years after the rhGH treatment, respectively [12], but the authors did explore the effect of hypopnea on the OAHI. A recent study has provided evidence indicating that there is a correlation between the aggravation of SRBDs and the progressive rise in insulin resistance among children with simple obesity and patients with PWS treated with rhGH [26].

To monitor the effect of rhGH treatment on SRBDs, PSG-associated indices were recorded before the treatment initiation, at 26 weeks and 52 weeks following rhGH treatment. At 26 weeks after rhGH treatment, OAHI, OAI, CAI, and ODI exhibited a decrease, indicating a potential reduction in the incidence of SRBDs in children with PWS receiving rhGH treatment, although statistical significance was not observed. Correspondingly, Festen et al*.* demonstrated that after 26 weeks of treatment of rhGH treatment in PWS children, the CAI and AHI levels decreased [27]. However, Miller et al*.* reported that 32% of the PWS patients experienced worsening of sleep disturbance in the presence of upper respiratory tract infection and adenotonsillar hypertrophy [28]. It is important to note that the limited sample sizes and broad age ranges in different studies may introduce potential selection biases.

In this present study, the mean value of SpO_2_ and the lowest SpO_2_ at 26 weeks and 56 weeks after initiating treatment exhibited no significant differences across different time points. More importantly, a notable finding was the decrease in the duration of SpO2 below 90% and the proportion of SpO2 below 90% at 26 weeks post-treatment initiation. However, compared to the 6-month time point, the duration of SpO2 below 90% increased at 52 weeks, although it remained lower than the onset of treatment. Additionally, the proportion of SpO2 below 90% also increased. At the 1-year time point, the value exceeded that of the rhGH treatment onset, but this increase was not statistically significant. In a study conducted by Zimmermann et *al*., they compared different onset age groups for rhGH treatment in PWS patients (younger than 1 year old or older than 1 year old) and found no significant differences in OAHI, CAI, ODI, and SpO2 depending on treatment onset [25]. While short-term rhGH therapy ﻿showed worsening ODI, the group treated with rhGH for a longer duration did not significantly differ from the control group [29].

IGF-1, a growth hormone with molecular similarity to insulin, plays a crucial role in mediating the anabolic effects of pituitary GH and linear growth through its interaction with insulin-like growth factor binding protein 3 (IGFBP-3) [30]. Patients with PWS are sensitive to rhGH treatment and usually have high levels of IGF-1 [31, 32]. IGF-1, as a major downstream target of GH, influences growth, development, and tissue homeostasis [7]. Nevertheless, elevated levels of IGF-1 have been associated with lymphoid hyperplasia, potentially increasing the risk of OSA and raising concerns of malignancy [33]. However, our study revealed that despite rhGH therapy leading to a significant increase in IGF-1 and IGFBP-3, this elevation did not contribute to the development of OSA. This finding aligns with the research conducted by Shukur et al., who observed a significant rise in IGF-1 levels among the rhGH-treated group without significant differences in respiration and sleep parameters [34]. While the association between IGFBP-3 and sleep-related breathing disorders (SRBDs) in PWS remains unexplored, our study indicates that the heightened levels of IGF-1 and IGFBP-3 resulting from rhGH treatment do not adversely affect SRBDs.

Consensus guidelines for rhGH treatment in children with PWS recommend conducting a baseline PSG evaluation before initiating treatment, followed by repeat assessments within the first 3–6 months. In cases where there is a deterioration of sleep-disordered breathing, snoring, or enlargement of tonsils and adenoids, additional evaluations such as ear, nose, and throat assessments, PSG, and IGF-1 measurements are deemed necessary [3].

Even though rhGH treatment did not exert any negative impact on SRBDs, several limitations were encountered in this study. Firstly, it is important to acknowledge that this analysis is a retrospective, non-randomized, single-institution study with a limited sample size. For further high-level investigations, we recommend a prospective, randomized, and multi-centre collaborative study to confirm the safety of rhGH treatment in toddlers. Secondly, this dataset only consisted of PWS patients with rhGH treatment and was limited to a 1-year follow-up period. To obtain a more comprehensive understanding of patient prognosis, a longer follow-up duration would be necessary. Lastly, it is noteworthy that adenotonsillar evaluation plays a pivotal role in the pathophysiological of SRBDs. However, regrettably, this aspect was not incorporated into our evaluations. Future studies should consider incorporating thorough adenotonsillar assessments to provide a more comprehensive analysis of SRBDs in relation to rhGH treatment.

## Conclusion

The administration of rhGH to children under 3 years of age with PWS for a duration of 52 weeks was found to be relatively safe, as evidenced by the absence of any toxic or adverse effects during the 1-year follow-up period. Importantly, rhGH treatment did not result in detrimental effects on SRBDs or their associated indices. Notably, the level of IGF-1 and IGFBP-3 increased following the initiation of the treatment, however, this elevation did not negatively impact respiratory and sleep parameters. This present study sheds more light on the clinical practice of rhGH treatment for patients with PWS.

### Supplementary Information

**Additional file 1: Supplementary Table1. **Comparison of the PSG assessments in the rhGH group and the non-rhGH treatment group.

**Additional file 2: Supplementary Table 2. **Comparison of the PSG assessments before and after rhGH treatment.

**Additional file 3: Supplementary Table 3.** Logistic regression of non-mild OSA relevant factors.

## Data Availability

All data used and/or analysed during the current study are available from the corresponding author on reasonable request.
