# Supplementary material for: Evaluating the effect of recombinant human growth hormone treatment on sleep-related breathing disorders in toddlers with Prader–Willi syndrome: a one-year retrospective cohort study
Source: BMC Pediatr. 2024 Jan 10;24:32. doi: 10.1186/s12887-023-04513-0 (PMC10777505; doi:10.1186/s12887-023-04513-0)
Supplement: Supplementary file 1 — Additional file 1: Supplementary Table1. Comparison of the PSG assessments in the rhGH group and the non-rhGH treatment group. [file 12887_2023_4513_MOESM1_ESM.docx]

Supplementary table1 Comparison of the PSG assessments in the rhGH group and the non-rhGH treatment group.

|  | non-rhGH (n=17) | rhGH (n=17) | *p* value |
| --- | --- | --- | --- |
| OAHI | 7.20±10.73 | 3.89±2.88 | 0.63 |
| OAI | 3.54±5.52 | 1.98±2.76 | 0.37 |
| CAI | 0.05±0.09 | 0±0 | 0.24 |
| ODI | 11.00±13.78 | 8.54±6.83 | 0.81 |
| Mean SpO2 | 95.91±3.56 | 95.97±2.38 | 0.47 |
| Lowest SpO2 | 82.71±11.25 | 76.64±14.60 | 0.27 |
| Time of SpO2<90% | 9.57±20.37 | 19.44±48.48 | 0.52 |
| Proportion of SpO2 < 90% | 4.28±8.41 | 5.45±12.03 | 0.61 |
