# Supplementary material for: Evaluating the effect of recombinant human growth hormone treatment on sleep-related breathing disorders in toddlers with Prader–Willi syndrome: a one-year retrospective cohort study
Source: BMC Pediatr. 2024 Jan 10;24:32. doi: 10.1186/s12887-023-04513-0 (PMC10777505; doi:10.1186/s12887-023-04513-0)
Supplement: Supplementary file 2 — Additional file 2: Supplementary Table 2. Comparison of the PSG assessments before and after rhGH treatment. [file 12887_2023_4513_MOESM2_ESM.docx]

Supplementary table2 Comparison of the PSG assessments before and after rhGH treatment.

|  | Pre-rhGH | Post rhGH(26w) | Post rhGH(52w) | *p* value |
| --- | --- | --- | --- | --- |
| AHI | 4.65±6.21 | 3.82±2.97 | 3.88±2.88 | 0.89 |
| OAI | 1.87±3.59 | 1.18±1.78 | 1.97±2.76 | 0.53 |
| CAI | 0.20±0.63 | 0.03±0.12 | 0.00±0.00 | 0.26 |
| ODI | 17.4±26.41 | 11.06±8.20 | 8.54±6.83 | 0.33 |
| Mean SPO2 | 96.45±4.87 | 96.05±1.77 | 95.97±2.38 | 0.46 |
| Lowest SPO2 | 77.35±8.89 | 78.00±9.53 | 76.65±14.60 | 0.14 |
| Time of SpO2<90% | 13.84±16.11 | 4.22±4.71 | 9.44±49.48 | 0.25 |
| Proportion of SpO2< 90% | 2.20±2.93 | 1.02±1.27 | 5.44±12.03 | 0.55 |
