# Supplementary material for: Evaluating the effect of recombinant human growth hormone treatment on sleep-related breathing disorders in toddlers with Prader–Willi syndrome: a one-year retrospective cohort study
Source: BMC Pediatr. 2024 Jan 10;24:32. doi: 10.1186/s12887-023-04513-0 (PMC10777505; doi:10.1186/s12887-023-04513-0)
Supplement: Supplementary file 3 — Additional file 3: Supplementary Table 3. Logistic regression of non-mild OSA relevant factors. [file 12887_2023_4513_MOESM3_ESM.docx]

Supplementary table3 Logistic regression of non-mild OSA relevant factors

|  | B | SE | Wald | *p* value | OR | 95% CI |
| --- | --- | --- | --- | --- | --- | --- |
| Obesity and overweight | 0.50 | 1.97 | 0.06 | 0.80 | 1.65 | 0.04-78.96 |
| rhGH | 0.68 | 1.67 | 0.17 | 0.68 | 1.98 | 0.07-52.55 |
| IGFBP-3 | -0.88 | 0.75 | 1.37 | 0.24 | 0.42 | 0.09-1.80 |
| W/H_z-score | -0.50 | 0.48 | 1.12 | 0.29 | 0.60 | 0.24-1.54 |
| IGF-1 z-score | 0.79 | 0.57 | 1.95 | 0.16 | 2.21 | 0.73-6.72 |
| genotype | -0.910 | 1.14 | 0.63 | 0.43 | 0.40 | 0.04-3.78 |
| Constant | 0.96 | 2.41 | 0.16 | 0.69 | 2.61 |  |
